# The Effects of Vitamin C on the Multiple Pathophysiological Stages of COVID-19

**DOI:** 10.3390/life11121341

**Published:** 2021-12-03

**Authors:** Jorge R. Miranda-Massari, Alondra P. Toro, Doris Loh, Jose R. Rodriguez, Raul Morales Borges, Victor Marcial-Vega, Jose Olalde, Miguel J. Berdiel, Neil H. Riordan, Juan Manuel Martinez, Armando Gil, Michael J. Gonzalez

**Affiliations:** 1 Medical Sciences Campus, School of Pharmacy, University of Puerto Rico, San Juan, PR 00936, USA; jorge.miranda2@upr.edu; 2 Program of Naturopathic Sciences, DP University, San Juan, PR 00918, USA; jrodriguez@albizu.edu; 3 Medical Sciences Campus, School of Public Health, University of Puerto Rico, San Juan, PR 00936, USA; alondra.toro1@upr.edu; 4 Independent Researcher, Marble Falls, TX 78654, USA; lohdoris23@gmail.com; 5 PhD Clinical Psychology Program, San Juan Campus, Carlos Albizu University, San Juan, PR 00901, USA; 6 Ashford Institute of Hematology & Oncology, Integrative Optimal Health of Puerto Rico, San Juan, PR 00907, USA; raul.morales.borges@gmail.com; 7 School of Medicine, Universidad Central del Caribe, Bayamon, PR 00960, USA; marcialvegamd@aol.com; 8 Centro Médico Regenerativo, Caguas, PR 00918, USA; joseolalde@gmail.com; 9 Berdiel Clinic, Ponce, PR 00717, USA; drberdiel@gmail.com; 10 MediStem Panama Inc., Panama City 32405, Panama; nhriordan@gmail.com; 11 Ortho-Regenerative Medicine, Chía 250001, Colombia; info@drjuanmanuelmartinezm.com; 12 Gil Pharmaceutical, Ponce, PR 00730, USA; agil3123@gmail.com; 13 School of Chiropractic, Universidad Central del Caribe, Bayamon, PR 00960, USA

**Keywords:** COVID-19, ascorbic acid, intravenous vitamin C, pathophysiology of COVID-19, Sars-Cov-2

## Abstract

Currently available anti-viral drugs may be useful in reducing the viral load but are not providing the necessary physiological effects to reduce the SARS-CoV-2 complications efficiently. Treatments that provide better clinical outcomes are urgently needed. Vitamin C (ascorbic acid, AA) is an essential nutrient with many biological roles that have been proven to play an important part in immune function; it serves as an antioxidant, an anti-viral, and exerts anti-thrombotic effects among many other physiological benefits. Research has proven that AA at pharmacological doses can be beneficial to patients with acute respiratory distress syndrome (ARDS) and other respiratory illnesses, including sepsis. In addition, High-Dose Intravenous Vitamin C (HDIVC) has proven to be effective in patients with different viral diseases, such as influenza, chikungunya, Zika, and dengue. Moreover, HDIVC has been demonstrated to be very safe. Regarding COVID-19, vitamin C can suppress the cytokine storm, reduce thrombotic complications, and diminish alveolar and vascular damage, among other benefits. Due to these reasons, the use of HDIVC should be seriously considered in complicated COVID-19 patients. In this article, we will emphasize vitamin C’s multiple roles in the most prominent pathophysiological processes presented by the COVID-19 disease.

## 1. Introduction

More than 100 million COVID-19 cases have been reported worldwide. This novel virus has caused a global health crisis. SARS-CoV-2 has three problematic characteristics. First, it seems to infect with a relative smaller viral load compared to other viruses, which makes it very contagious. Second, SARS-CoV-2 mutates fast, which can make available emergency vaccines less effective against new emerging strains, such as the Delta variant, which is characterized for manifesting more severe symptoms and being even more contagious than the original strain. Third, this virus causes a dangerous inflammation response that generates numerous free radicals and inflammatory molecules that can be highly cytotoxic and damaging. Meanwhile, hospitals have been treating infected patients with anti-viral drugs, such as remdesivir, hydroxychloroquine, and lopinavir. A recent study concluded that these drugs appeared to have little or no effect on hospitalized COVID-19 patients [1]. These and other reasons should ignite our interest to keep searching for treatments that can activate efficiently systemic defenses in order to generate better clinical outcomes for complicated COVID-19 patients.

One of the most important aspects to combat the SARS-CoV-2 is to have an optimized immune system that works properly and efficiently. In order for this system to function well, it needs a wide range of specific cofactors. One of these cofactors is vitamin C, also known as ascorbic acid. This powerful, water-soluble antioxidant is involved in many biological processes of the immune response [2]. Furthermore, this vitamin has shown potent anti-viral and anti-inflammatory activities in a variety of different viral infections [3,4]. Although oral vitamin C produces adequate concentrations to produce physiologic effects, pharmacologic concentrations require high intravenous doses that may be able to produce therapeutic benefits to COVID-19 patients.

Vitamin C is part of a comprehensive strategy to COVID-19. An ambulatory early management protocol of COVID-19 from an international consensus of experienced clinicians [5] proposed a multifaceted highly targeted sequential multidrug treatment that include vitamins, minerals, antimicrobials, steroids, colchicine, and possibly antithrombotic agents to assist the body deal with the viral load and the possible inflammatory and complications. A study in patients following this protocol demonstrated that early ambulatory treatment resulted in 87.6% reduction in hospitalization and 74.9% reduction in deaths [6].

Intravenous vitamin C has been successfully used in the hospitalized COVID-19 protocols [7]. It has been suggested that since vitamin C has both anti-inflammatory and antiviral effects, its use may help reduce drug dosing and toxicity [8]. Vitamin C, when given in high doses, is even extremely safe [9], and it can be easily excreted through the urine.

In this article, we will emphasize how vitamin C can attenuate many of the pathophysiological stages presented by the SARS-CoV-2 infection. Additionally, based on this information, we suggest that the administration of adequate dose of intravenous vitamin C combined with proven conventional supportive medications could provide COVID-19 patients with better clinical outcomes due to the multiple beneficial physiological properties it can exert. Moreover, AA is relatively non-toxic, and side effects are minimal.

Although oral intake of vitamin C does have a role in health maintenance, risk reduction and prevention, and prevention of infections, this article is focused on the more potent effects that can be achieved with pharmacologic concentrations.

## 2. High-Dose Intravenous Vitamin C (HDIVVC): Its Relevance

There are multiple ways vitamin C can be administered. It can be given either orally or intravenously. However, both methods have different physiological effects. In particular, there are studies that established differences between oral and IV vitamin C [10]. It was demonstrated that blood levels of vitamin C in patients were much higher by IV vitamin C than oral dosing. Because IV vitamin C is 100% bioavailable, it has the capacity to replenish tissues more efficiently and rapidly. Oral vitamin C at single doses of 200 mg is complete, and Cmax is about 60 min. However, it must be noted that under normal conditions in a healthy subject, bioavailability is reduced, which means that, for example, only about 33% a dose of 1250 mg or 412.5 mg will be absorbed when using a regular vitamin C formulation [11,12]. The decreased bioavailability of the larger oral doses is caused by saturation of the absorption mechanisms [13]. The oral dose must be absorbed by the small intestine, while IV vitamin C bypasses this route and is more readily available. Therefore, it is important to state that, to have better physiological effects that may lead to better clinical outcomes, IV vitamin C is the most potent method to attain higher blood concentrations. Another important aspect to highlight is that the effect of vitamin C will depend on frequency of application and quantity given. Multiple reports providing higher doses of IV vitamin C in a range from 30 to 150 g showed beneficial effects in cancer patients [14]. Moreover, in addition to all the benefits that high-dose IV vitamin C exerts, it is important to point out that it is very safe and non-toxic. Recently, there was a study published using high-dose IV vitamin C on COVID-19 patients [15], and results showed multiple improvements but, most importantly, no adverse effects.

Although there is no consensus about how to classify the doses in terms of magnitude, for the purpose of this article, we will refer to intravenous vitamin C in the context of a pharmacological effect and therefore will consider a low intravenous total daily dose to be 6–12 g, moderate intravenous total daily dose to be 13–24 g, and high dose intravenous total daily dose to exceed 25 gm daily.

In conclusion, high-dose IV vitamin C will provide better physiological effects leading to improved clinical outcomes due to higher concentrations while being safe and non-toxic.

## 3. Anti-Viral Mechanisms of Vitamin C

As mentioned previously, vitamin C is an essential nutrient for the body with several beneficial properties that help support proper functioning of the immune system, of great interest being its anti-viral capacity. Vitamin C has direct and indirect mechanisms that can exert these anti-viral properties. It has been shown that vitamin C can inactivate in vitro a wide range of viruses [16]. Virus inactivation was shown to be dependent on oxygen and was concluded to be mediated through oxidation to viral nucleic acids [17]. There is a possibility that ascorbate may damage viral capsids and even inhibit viral replication when provided in large doses. An in-vitro study showed that pharmacological ascorbate killed influenza virus in cultured human bronchial epithelial cells [18].

On the other hand, an indirect mechanism was proven by multiple studies in which vitamin C exerts powerful antiviral activity [19]. In a clinical study of 178 patients with Epstein Barr viral (EBV) infection treated with high doses of intravenous vitamin C, an inverse correlation was found between EBV viral capsid antigen (VCA), IgM, and vitamin C in plasma in patients with mononucleosis and chronic fatigue syndrome. Patients with high levels of vitamin C had lower levels of antigens in the acute state of disease [20].

Vitamin C can promote the production of anti-viral proteins, such as interferon. In an in-vitro study, there was evidence that, in the presence of ascorbic acid plus glutathione, interferon was produced [21]. These proteins play a role in immune protection and interfere with viral replication by binding to the cell surface. Moreover, a study analyzed in a mice model the multiple effects model of high-dose oral vitamin C supplementation the initial stage of influenza A virus (H3N2) [22]. They concluded that ascorbic acid could exert anti-viral activity by increasing the production of interferon-α/β. In addition, an animal study supports additional antiviral mechanisms of vitamin C. The administration of high doses of vitamin C caused virus attenuation of H1N1 virus. This study also showed that the intervention produced decreased expression of susceptibility genes and increased production of NF-κΒ, leading to the production of type I interferon (IFNs) [23]. Additionally, ascorbic acid enhanced the interferon levels produced by human embryo skin and human embryo lung fibroblasts induced by Newcastle disease virus [24].

## 4. Clinical Reports

### 4.1. Non-COVID Viral Infections

There are several studies reporting good clinical outcomes employing IV vitamin C on patients infected with different types of viruses. In example, there was a case report of a 54-year-old patient with chikungunya (CHIKV) fever with severe joint pain and inflammation [25]. The infected patient was treated with high doses of IV vitamin C for two days. Symptoms resolved promptly without any side effects during or after the infusions. In another case report, a 25-year-old patient tested positive for influenza virus [26]. Supplementation of IV vitamin C was given, resulting in fast improvement and returning to normal on day four. Therefore, these cases highlighted the effectiveness of IV vitamin C against combating viral infections.

Vitamin C has demonstrated that it can inhibit manifestations of viral ARDS. A case report showed how high doses of IV vitamin C can help stabilize a patient and help with decreasing the recovery time [27].

Later, there was a trial with patients suffering from sepsis and ARDS [28], in which vitamin C infusions were administered. Interestingly, results showed that vitamin C decreased mortality and length of stay for infected patients. In addition, there was a case report of a woman suffering with ARDS and other complications, in which the patient was intubated, presented hypoxemia, and chest imaging revealed opacities. Despite various approaches, the patient remained critical. Once IV vitamin C was initiated, the patient’s chest imaging and oxygenation began to improve. Rapidly, the woman became stable [29].

Some recent studies suggest that the use of ascorbic acid as part of a protective protocol against SARS-CoV-2 can reduce mortality in patients hospitalized in intensive care units with sepsis. This ascorbic acid protocol can significantly reduce mortality from sepsis [30], reduce ICU stay length, and significantly reduce the time to resolution of shock [31]. The use of ascorbic acid injections also improved the ventricular function (EF) 72 h after surgery and reduced the length of ICU stay in patients undergoing coronary artery bypass surgery.

### 4.2. COVID Viral Infections

The proven ability of vitamin C against other types of viruses may very well be equally as effective in SARS-CoV-2. The following cases support this this premise:

1. A case report of a COVID-19 patient with early use of high-dose intravenous vitamin C (HDIVC) showed positive outcomes [32]. The patient presented with chest X-ray opacities and infiltrations, body pain, dry cough, and other symptomatology. As she tested positive for SARS-CoV-2, 25 g of vitamin C was administered once a day for three consecutive days. The following day of the first infusion, the patient noted a dramatic improvement. Her body pain and headache were gone. Additionally, the most important aspect to highlight is that the infusions did not cause any adverse effects to the infected patient.
2. In an unusual early recovery case of a critical COVID-19 patient given vitamin C [33], a 74-year-old woman presented with fever, cough, and shortness of breath, oxygen saturation of 87%, and bilateral rhonchi. Chest radiography was suspicious for pneumonia and test was positive for SARS-CoV2. The patient was initially started on oral hydroxychloroquine and azithromycin. On day six, she developed ARDS and septic shock, for which mechanical ventilation and pressor support were started, along with infusion of high-dose IV vitamin C (11 g/d). The patient improved clinically and was able to be taken off mechanical ventilation within five days. This report emphasizes the potential benefits of high-dose IV vitamin C in critically ill COVID-19 patients in terms of speedy recovery and reduced length of mechanical ventilation and ICU stay. In this case, ascorbate seems to attenuate lung injury produced by the viral infection. It has been seen that even though a COVID-19 patient had already presented complications and developed critical conditions, such as acute respiratory syndrome ARDS or DAD, high doses of vitamin C can help stabilize, improve patient condition, and even shorten the length of the disease caused by the SARS-CoV-2 virus.
3. A case series of 17 high-risk patients with advanced age and multiple comorbidities who tested positive for COVID-19 and had moderate to severe disease were treated with IV vitamin C (1 g every 8 h for 3 days) in addition to standard treatment for COVID-19 [34]. Results showed a significant decrease in inflammatory markers, including ferritin and D-dimer, and a trend to decreasing FiO2 requirements. This case series presents a 17.6% rate of need for mechanical ventilation, which is comparable to a recent study, and 12% mortality rate, which is smaller [35]. Despite a relatively low dose of IVC, it produced comparable outcomes with some possible improvements.
4. In the case of a 77-year-old female with a history cardiovascular disease that presented with a five-day history, worsening symptoms received a variety of antimicrobials, anti-inflammatory agents, monoclonal antibodies, and IV vitamin C (6 g bid). She did not respond and eventually succumbed. This case illustrates the importance of early treatment to avoid the complications, such as cytokine storm, especially in patients with significant comorbidities. Once the cytokine storm manifests, even with aggressive and comprehensive treatments, it seems the body is unlikely to recover [36].
5. In this case report, two patients were confirmed with SARS-Cov2 pulmonary infection with respiratory distress at ages 57 and 58 years old, with 6–7 days of symptoms. Patient prior medical history was not reported and presumably had no risk factors. They were treated with oxygen in one case and mechanical ventilation in the other. Medications included hydroxychloroquine (600 mg/day), azithromycin, steroids, vitamin C (3 g/day), zinc, diuretics, and enoxaparin, which were equally used for the two patients. Both patients responded to treatment and recovered. The treatments described started on day 6–7 of symptoms but is unclear if patients had previous treatment before admission. In conclusion, this report is not helpful to assess the utility of the vitamin C in COVID-19 because the vitamin dose used were low. In addition, the route of administration is not specified as well as the presence of risk factors or previous therapy before hospitalization [37].
6. In a retrospective case series, twelve patients were enrolled, including six severe and six critical patients. All patients received high-dose intravenous vitamin C (average 163 mg/kg in severe patients), but on average, about a 10% higher dose was given to critical patients. Patients had significant improvements in CRP, lymphocyte count, and CD4. Improvements in oxygenation and SOF score were also observed. Greater improvements were observed in severe than critical patients after HDIVC. It was concluded that high-dose IVC (11 g per day average or more for a 70-kg person) could be beneficial in aspects of inflammatory response and immune and organ function for treatment of COVID-19 patients [38].
7. A retrospective case-matched clinical study compared the outcome and clinical courses of patients with moderate COVID-19 patients treated with an HDIVC protocol (100 mg/kg/day) for seven days from admission with a control group treated without the HDIVC. The HDIVC and control groups each comprised 55 patients. For the primary outcomes, there was a significant difference in the number of patients that evolved from moderate to severe type between the two groups. There was a significant reduction in the number of patients in the HDIVC that evolved from moderate to severe disease (*p* = 0.03). Additionally, compared to the control group, there was a shorter duration of systemic inflammatory response syndrome (SIRS, *p* = 0.0004) and lower SIRS occurrence (*p* = 0.0086) during the first week. It was concluded that in this population, a dose of 7 g for a 70-kg person may be of value in preventing progression from moderate to severe disease [39].
8. In an open-label, randomized, and controlled trial on patients with severe COVID-19 infection, the case and control treatment groups each consisted of 30 patients. The control group received lopinavir/ritonavir and hydroxychloroquine, and the experimental group received IVC (6 g daily) added to the same regimen. The experimental group receiving IVC showed significant improvements in mean body temperature, which was significantly lower on the third day of hospitalization (*p* = 0.001) and peripheral capillary oxygen saturation (SpO_2_) (*p* = 0.014). However, the median length of hospitalization was significantly longer than the control group (8.5 days vs. 6.5 days) (*p* = 0.028). There was no significant difference in the length of intensive care unit (ICU) stay and mortality between the two groups. It is noticeable that there were significant clinical differences in the initial response, and it seems that 6 g was not enough to change the clinical outcomes [40].
9. Finally, a recent placebo-controlled pilot study of high dose intravenous ascorbate in 56 critically ill COVID-19 patients showed significantly reduced mortality. The trial was conducted in two hospitals located in Wuhan, China, and used a daily dose of 24 g of ascorbate divided in two doses. The ICU mortality rate was 22% (6 out of 27) in the ascorbate group and 38% (11 out of 29) in the placebo. This difference did not reach statistical significance because of the small number of patients. However, in the most critically ill (SOFA > 3), the difference in hospital mortality was even bigger: 18.5% in the ascorbate group vs. 38% in placebo, achieving statistical significance (*p* = 0.04) [41].

In summary, when examining the existing literature for the use of intravenous vitamin C in COVID-19 patients, there is consistence in the evidence of improvements in outcomes, such as fever, inflammation, cell blood count, oxygenation, and even clotting markers. The case studies and small studies discussed provide some indication that the time to begin treatment, the dose of vitamin C, the presence of comorbidities, and the severity all play a role in response and mortality. However, it seems that better outcomes are associated with higher doses.

## 5. Vitamin C Suppresses Oxidative Stress

Oxidative stress is an imbalance of antioxidant molecules and free radicals species in the body. This can cause damage of DNA, membranes, proteins, and others, which can result in complications and diseases [42]. These free radicals are also referred to as oxygen reactive species (ROS). Exhibiting low concentrations of ROS and having an adequate homeostasis of them is beneficial for the body since they are needed in the immune system to fight pathogens, such as viruses. However, in high concentrations, these reactive molecules are toxic and may exert very damaging effects. It has been observed that when there is a viral infection, viruses can induce via multiple pathways severe oxidative stress [43]. Viruses are often associated with oxidative stress that can result in RedOx imbalances that can cause damage to cells. Regarding coronaviruses and SARS-CoV-2, increased levels of oxidative stress in infected patients seems to have a detrimental effect in cells and organs [8].

Some studies have proposed that a precise disulfide-thiol balance is crucial for viral entry and fusion into the host cell and that oxidative stress generated from free radicals can affect this balance. It seems possible that oxidation of thiols to disulfides, under a mechanism of oxidative stress, would improve the affinity of SARS-CoV-2 S proteins for the ACE2 receptor and consequently increase the severity of COVID-19 infection [44]. Oxidative stress refers to the harmful effects from excessive free radicals or prooxidants in a biological system. Advanced age is a risk factor for COVID-19 morbidity and mortality. Age-related decline GSH systems and the impaired control of the thiol to disulfide balance alters RedOx balance of all tissues. Using a specialized computational system for molecular dynamics simulation and for binding energy calculations, it was determined that the binding affinity was significantly impaired when all the disulfide bonds of both ACE2 and SARS-CoV/CoV-2 spike proteins were reduced to thiol groups. If this was the case, providing the body with increased glutathione precursors and cofactors, as well as electron donors like vitamin C, which regenerates reduced glutathione, would result in decreased entrance of the virus, reduced viral load, and consequently reduced severity of the COVID-19 infection [45,46].

In addition, an association of oxidative stress in COVID-19 and the amplification and perpetuation of the cytokine storm, coagulopathy, cell hypoxia, and mitochondrial dysfunction has been shown, suggesting a possible therapeutic role of antioxidants and other agents to reduce oxidative stress [47].

There was a study on COVID-19 patients evaluating levels of antioxidants and oxidative stress markers that concluded that infected patients had significantly lower levels of antioxidants [48]. Moreover, they stated that severe COVID-19 patients are at higher risk of oxidative stress.

Although COVID infection is the likely cause of depleted antioxidant cofactors, that study was not designed to determine cause and effect.

The NHANEs has established that there is a significant proportion of the USA population deficient in nutrients, including vitamin C (46%), vitamin A (45%), vitamin E (84%), and Vitamin D (95%) [49]. In a study with five hospitalized patients with COVID-19, researchers observed a deficiency of vitamin D in 76% and of selenium in 42% of the patients [50]. Although vitamin D is not considered and antioxidant vitamin, vitamin D reduces renin-angiotensin-aldosterone system activation and consequently decreases ROS.

Activation of the renin-angiotensin-aldosterone system and consequent increase in angiotensin II and aldosterone, as seen in cardiometabolic syndrome, participates in altering insulin/IGF-1 signaling pathways and reactive oxygen species formation to induce endothelial dysfunction and cardiovascular disease [51]. The mechanisms involved in these inhibitory effects of ANG II include the generation of ROS [52].

An animal study in a model of hyperoxia-induced acute lung injury (HALI) found that 50 mg/kg of parenteral vitamin C produces a significant decrease in the levels of airway HMGB1 in hyperoxia control (*p* < 0.05), leukocyte infiltration (*p* < 0.05), and improved lung integrity in the animal treated with AA [53]. This study, while not related to a viral infection, demonstrated the role of high-dose vitamin C in reducing damage from oxidative stress, inflammation, and lung integrity, which are pathophysiologic processes relevant to COVID.

Since COVID-19 patients commonly have lower levels of vitamin C due to the physiological stress of the viral infection [54], replacement of this vitamin in the optimal amounts to control oxidative stress as well to other benefits for COVID-19 patients should be considered. While low vitamin C status is associated with severe COVID, we do not yet know if patients with low vitamin C levels (pre-infection) are at greater risk of developing severe COVID or if severe COVID infections cause people to lose vitamin C more quickly. While both are likely, knowing this could assist in promoting preventive and health promotion measures.

## 6. Vitamin C Inhibits the Cytokine Storm Due to Its Antioxidant Capacity

The severity of being infected by SARS-CoV-2 is related to the cytokine storm, which can occur in many tissues. In the lungs, and in particular the alveoli, it can cause a proinflammatory response that leads to pneumonia, ARDS, diffuse alveolar damage (DAD), multiorgan failure, and other complications. Studies in Wuhan, China, showed that patients with severe COVID-19 symptoms had higher levels of IL-6 and other inflammatory cytokines in their blood samples [55]. It is normal for the body to increment the secondary (humoral) immune response after a high viral load exposure, which subsequently causes the release of these inflammatory cytokines that attack foreign proteins, such as those presented by SARS-CoV-2. However, it is expected that the body itself will gradually lower the immune response. In COVID-19 patients, the immune response does not seem to lower, causing detrimental and fatal effects to many organs. Due to this, the role of the cytokine storm rampage seen in COVID-19 has generated interest from the medical-scientific community as a potential target for combating the SARS-CoV-2 complications that may lead to death.

Furthermore, the alveolus is a structure that is very much affected by the cytokine storm. A common finding of post-mortem autopsies from COVID-19 patients reveals a trend of patients with highly damaged alveolar structures. A study noted that all of the seven COVID-19 lung specimens observed had diffuse alveolar damage (DAD) [56]. Another study observing pathogenesis of two severely infected COVID-19 patients found that the DAD seen in acute respiratory distress syndrome (ARDS) was not unique to COVID-19 patients but occurs in both SARS and MERS infections as well [57], which indicates that ARDS may be responsible for the DAD observed in COVID-19 cases. Furthermore, a third study stated that the development and progression of ARDS seen in COVID-19 is closely related to the inflammatory cytokine storm [58]. Outlining this information, suppression of the cytokine storm is of particular interest because it can prevent further complications, such as DAD and ARDS. Moreover, if a treatment can decrease the possibilities of the generation and/or progression of the cytokine storm, it could greatly decrease the possibilities of mortality in COVID-19 patients since they are highly correlated [59].

Vitamin C can inhibit the cytokine storm in COVID-19 patients based on its antioxidant properties. The main concern of the cytokine storm is the inflammatory response it produces, and one of the principal functions of this vitamin in addition to its great antioxidant capacity is that it exerts anti-inflammatory properties. It has already been observed in human and animal models that high doses of vitamin C may decrease several inflammatory parameters, such as inflammatory cytokine release and activity. For example, it was discovered that when a high dose of IV vitamin C was given to critically ill COVID-19 patients, their levels of interleukin-6 (IL-6) were lower than the placebo group (Jing et al., 2020). In addition, there is evidence that vitamin C inhibits a variety of cytokines [60], A group of 12 cancer patients receiving 25–125 gm of intravenous vitamin C and achieving 5–18 mM of ascorbate plasma concentration experienced normalization of many of the cytokine levels measured. Cytokines that were most consistently elevated prior to treatments included M-CSF-R, leptin, EGF, FGF-6, TNF-α, β, TARC, MCP-1,4, MIP, IL-4, 10, IL-4, and TGF-β. Cytokine levels tended to decrease during the course of treatment.

The intense proinflammatory response to the SARS-CoV-2 infection can lead to the development of a life-threatening cytokine release syndrome (CRS), which can lead to acute respiratory syndrome (ARDS), leading to a high mortality rate in elderly subjects and other at-risk populations. Furthermore, vitamin C supplementation can generate stable, antigen-specific regulatory T cells in animal models of autoimmune or acute graft versus-host diseases. Vitamins may shift the proinflammatory T-helper lymphocytes (Th17)-mediated immune response arising in autoimmune diseases towards a T-cell regulatory phenotype [61,62].

On the other hand, some reviews postulate that vitamin C can help remove alveolar fluid caused by distress of the inflammatory response [63]. Parenteral vitamin C infusion protected mice from the harmful consequences of sepsis by several mechanisms, including attenuation of the proinflammatory response, enhancement of epithelial barrier function, increasing alveolar fluid clearance, and prevention of sepsis-associated coagulation abnormalities [64]. An in-vitro study found that vitamin C acts at multiple levels to exert its antiviral and protective functions in the lungs significant upregulation of several metabolic pathways and interferon-stimulated genes (ISGs) along with a downregulation of pathways involved in lung injury and inflammation [65].

Therefore, AA is promising for preventing and treating the cytokine storm itself.

## 7. Vitamin C Lessens Alveolar Damage and Lung Complications

One area that SARS-CoV-2 is likely to invade is the lung tissue, and a correlation has been observed between the virus and alveolar damage. It is very common for infected patients to develop ARDS, which is associated with large amounts of inflammation and damage on the alveolar-capillary barrier. Additionally, infected COVID-19 patients suffer further complications, resulting in permanent alveolar damage. When an infected patient has developed complications, such as ARDS and DAD, there is higher chance of mortality, and prognosis may seem to worsen. For instance, it has been established that ARDS is one of the principal causes of high mortality in patients with COVID-19 [66]. In order to reduce mortality rates and provide the patient with better clinical outcomes, it is important to identify better treatment that can directly manage these complications.

A brief research letter reported that vitamin C levels were undetectable in more than 90% of COVID-19 patients suffering with ARDS [67]. In addition, an epidemiological study reported that up to 82% of critically ill COVID-19 adult patients with ARDS had low vitamin C values [68].

This is consistent with low plasma levels of vitamin C reported in critically ill patients and many other conditions. Septic shock patients have significantly depleted vitamin C levels compared with non-septic patients, likely resulting from increased metabolism due to the enhanced inflammatory response [69,70]. Low vitamin C levels are also observed in patients with infections [71], trauma patients [72], cancer patients [73], and diabetes patients [74]. Patients with these complications have low plasma levels and benefit from high quantities of vitamin C to efficiently compensate from its excessive utilization by their immune system due to an increased physiological stress. Therefore, the supplementation of IV vitamin C can be beneficial to help maintain optimal protective levels in these patients and consequently maintain cellular homeostasis.

Although more research needs to be done in order to fully validate the effect IV vitamin C has on ARDS, current information seems very promising and appears to exert many beneficial properties on patients with ARDS. Some of the cases were discussed previously in Section 4.

## 8. Vitamin C Suppresses Thrombosis and Prevents Vascular Tissue Damage

Current research has been stating that SARS-CoV-2 may predispose infected patients to thrombotic diseases causing alteration in the coagulation cascade and leading to a worse prognosis [75,76]. Thrombotic complications could also significantly contribute to mortality. Furthermore, the COVID-19 disease can have a prothrombic state, and it can be seen in multiple organs, such as lungs, spleen, and gut [77]. This dangerous complication is due to the excessive performance of the immune system trying to combat the virus, which generates significant amounts of clotting substances in the blood. It can be said that inflammation and coagulation are interrelated when we speak about SARS-CoV-2. Currently, one of the typical ways to treat these thrombotic complications on COVID-19 patients are blood thinners. However, these medications do not always prevent clotting in infected patients, and there are minimal studies proving reliability [78]. In addition, a study highlighted that despite administration of anticoagulation medication, COVID-19 patients continued to have thrombotic complications [79]. This coagulopathy described in COVID-19 is characterized by an increase in procoagulant factors, such as fibrinogen and D-dimers, which have been associated with higher mortality.

Moreover, another structure that seems damaged by this virus are the endothelial cells found in the blood vessels. Studies show that particles from SARS-CoV-2 and inflammatory cells were detected in endothelial cells of infected patients [80]. This could be directly correlated to the thrombotic complications since blood vessels require healthy endothelial cells in order to inhibit the formation of clots. Moreover, when a virus is present, it can provoke the phagocytosis of important proteins from cells such as endothelial cells, causing loss of vascular integrity, cessation of important anti-clotting reactions, and leading to severe complications.

One of the many properties of vitamin C is that it exerts an antithrombotic action. It could be beneficial for COVID-19 patients to receive IV vitamin C to prevent or decrease thrombotic complications. Moreover, a study concluded that providing vitamin C during early stages of the disease prevented coagulopathy and the formation of microthrombi, which are common complications of COVID-19 [81]. Another important research to highlight is IV vitamin C and evaluation of D-dimer levels. The D-dimer test is a blood test used to detect presence of blood clots. Clinicians constantly order this test since it has been proven to be the best laboratory diagnostic marker for COVID-19-associated hemostatic abnormalities and is directly related with mortality [82]. Recently, there was a study of 17 COVID-19 patients that were given IV vitamin C [34]. Results showed that not only did vitamin C decrease the mortality rate but some inflammatory markers, such as D-dimer levels, were also decreased.

Nearly 40% of the protein content in the body is composed of collagen [83]. Collagen is a molecule that plays an important role in the health of the vascular system. This protein strengthens and protects the integrity of blood vessels. Each blood vessel contains a layer of endothelial cells that contain and need collagen to work properly. Collagen polypeptide synthesis, posttranslational hydroxylations, and actions of the two hydroxylases are independently regulated by vitamin C [84]. Vitamin C also increases the synthesis and deposition of type IV collagen in the basement membrane, stimulating endothelial proliferation [85], promoting adhesion [86], inhibiting apoptosis [87], scavenging radical species, and sparing endothelial cell-derived nitric oxide to help modulate blood flow [88]. Since moderate and severe SARS-CoV2 infection produces high level of oxidative stress in many tissues, it therefore creates a higher demand for vitamin C since vitamin C insufficiency among healthy people in USA is 45% [49], it must be presumed that the vast majority of COVID-19 patients are depleted; however, for optimal collagen synthesis, oral might be enough. Although it is unknown if collagen synthesis is a likely path to influence COVID response in the short term or in the long term, this mechanism is likely to have an impact in a matter of days.

In addition to those involved in collagen synthesis, there is increasing evidence that ascorbate is required for optimal function of many dioxygenase enzymes. Several of these enzymes modulate the transcription of proteins involved in endothelial function, proliferation, and survival, including hypoxia-inducible factor-1α and histone and DNA demethylases. Additionally, vitamin C has been found acutely able to tighten the endothelial permeability barrier [89] and thus may modulate access of ascorbate and other molecules into tissues and organs [90].

Moreover, functional endothelial cells have the capacity of exerting antithrombotic properties and also possess the ability to degrade clots, preventing thrombosis [91]. Therefore, having healthy functional blood vessels can contribute to prevent thrombotic complications in patients with COVID-19.

## 9. Vitamin C Protects Red Blood Cells and Averts Hemoglobinopathy and Iron Metabolism Dysregulation

The Fenton reaction results from hydrogen peroxide (H_2_O_2_) catalyzed by iron (II) (Fe^2+^) to form hydroxide (OH^−^) and hydroxyl radical. Since the use of intravenous vitamin C at high doses promotes the formation of peroxide [92], the formation of hydroxyl radicals is increased. Human and mammal cells produce SOD, catalase, and peroxidases that can quickly reduce peroxide, therefore reducing damaging effects. Catalase is the most effective catalyst for the decomposition of H_2_O_2_ and is an abundant antioxidant enzyme commonly found in the liver, erythrocytes, and alveolar epithelial cells [93]. However, many cancer cells [94] and some bacteria and viruses lack this mechanism and are therefore vulnerable to the oxidative stress generated by this reaction. Obligate anaerobes usually lack all three enzymes. Oxidizing disinfectants, such as a 0.5% hydrogen peroxide solution, have been shown to be inactivate coronaviruses when used as a surface disinfectant [95]. An in-vitro study showed that catalase pathways are critically involved in intracellular ROS regulation, including the expression of the tumor suppressor gene p53 [96].

COVID-19 captures iron and generates reactive oxygen species to damage the human immune system. SARS-CoV-2 may also affect red blood cells and hemoglobin. Multiple proteins from SARS-CoV-2 can affect the hemoglobin pathway and cause dissociation of iron. In summary, the virus inhibited the immune system through by excessive ROS production [97]. SARS-CoV-2 proteins can interact with hemoglobin and cause denaturation of the molecule. These alterations could inhibit the ability of red blood cells to transport oxygen, leading to hypoxia. Moreover, the hemoglobin pathway can also be altered by the condition of ARDS, which is very common in COVID-19 patients. For example, it has been observed that patients with ARDS can have higher levels of cell-free hemoglobin (CFH) [98]. Having high levels of CFH can be detrimental since it can cause inflammation and alveolar injury [99]. In addition, cell-free hemoglobin has the unstable ferric form, and therefore, it can initiate a cascade of free radical chain reactions, causing more oxidative stress toxicity and tissue damage [100].

This hemoglobin pathway irruption caused by the virus or ARDS can worsen the respiratory condition in COVID-19 patients since the virus already compromises the respiratory system. In COVID-19 patients, if there is a decrease in functional hemoglobin, it can result in poor clinical outcomes [101]. Taking this into account, evaluations on how to decrease or inhibit the capacity of the virus to invade this pathway or affect the red blood cells in order to maintain better oxygenation levels and overall stability in patients should be considered.

Hemoglobin desaturation accompanied by iron metabolism dysregulation favors the following pathological pathways: hypoxemia and systemic hypoxia, reduction of nitric oxide, coagulation activation, ferroptosis with oxidative stress and lipid peroxidation, mitochondrial degeneration, and apoptosis [102]. This hemoglobin alteration can contribute to the oxygen-deprived multifaceted syndrome seen in many COVID-19 patients. Interestingly, the erythrocyte presents other SARS-CoV-2 receptors in addition to the ACE2, such as cyclophylins, furins, and TMPRSS2 [103].

Given the dynamics of hemoglobin during SARS-CoV-2 infection, it is important to mention that vitamin C is implicated in iron homeostasis. Vitamin C stimulates iron sequestration/removal from plasma and works with the haptoglobin system to limit the extent of damage after infection. Iron metabolism is modulated by ascorbate by stimulating ferritin synthesis, inhibiting degradation of lysosomal ferritin, and decreasing cellular iron efflux. Moreover, ascorbate cycling across the plasma membrane is the mechanism for ascorbate-stimulated iron uptake from low-molecular-weight iron-citrate complexes, which are important in the plasma of individuals with iron-overload disorders [104].

Silent hypoxia is described in COVID-19 patients, who show a progressive worsening hypoxia associated with normal CO_2_.This condition worsens in later stages, when CO_2_ increases. Finally, hyperferritinemia can increasingly affect alveolar/capillary cell membrane integrity/permeability, causing inflammation, edema, and lung necrosis and further complicating the pulmonary condition. Furthermore, hyperferritinemia may induce a series of injuries to many organs via autoimmunity, such as coagulopathies, macrophage activation syndrome, hemochromatosis like liver damage, and other ferroptosis-driven syndromes.

As for vitamin C, it is known that it can maintain hemoglobin iron complex in the ferrous state that allows binding of oxygen, and it can also improve heme iron/cell RedOx balance [102]. Vitamin C can indirectly decrease furin cleavage and reduce endothelial permeability to free heme [105]. Additionally, as mentioned earlier, vitamin C has antioxidant capacities that could help in managing the oxidative radicals produced by the cell-free hemoglobin. Lastly, the inflammation linked to high levels of CFH can be possibly managed by vitamin C due to its anti-inflammatory capacity. Vitamin C is required in high amounts in tissues to maintain vascular strength and integrity. The microvasculature, when depleted of vitamin C, loses its structural capacity, as shown in patients suffering scurvy. These derangements can further enhance coagulability issues and cause patients to be prone to both venous and arterial blood clots. Vitamin C improves the condition of critically ill complicated COVID-19 patients by its multiple beneficial physiological effects, which include attenuation of lipid peroxidation, reduction of vascular permeability, lessening microvascular dysfunction, preserving the endothelial function and microcirculatory flow, improving endogenous vasopressor synthesis, and increasing vasopressor sensitivity and hemodynamic stability, which ultimately leads to reduced organ injury and dysfunction that may save lives among the critically ill [106].

## 10. Vitamin C Protective Role on SARS-CoV-2 Multiple Organ Manifestations

It is known that lung tissue is the initial and primary target of invasion by the SARS-CoV-2 virus. However, is not uncommon for SARS-CoV-2 to invade other organs. It will all depend on the quantity and origin of receptors that each infected patient possesses. Increased research reveals that COVID-19 patients commonly exhibit multiorgan manifestations. For example, there have been reported cases of patients presenting kidney injury, gastrointestinal symptoms, cardiovascular issues, liver problems, and other complications [107]. In this sense, COVID-19 can be considered as a systemic disease. Additionally, research highlighted and observed via data analyses shows that multiple organs and tissues were vulnerable to SARS-CoV-2 virus and not only the lungs [108].

The renal system seems to be one of the most frequently targeted areas for SARS-CoV-2; in addition, autopsies have demonstrated that there is a percentage of COVID-19 patients that suffered from renal tropism [109]. This includes patients with or without kidney diseases. The study also highlighted that the presence of SARS-CoV-2 RNA in the kidney was associated with increased risk of premature death. Taking this into account, prevention and reduction of kidney injury or complications in order to decrease disease severity and provide better clinical outcomes in COVID-19 patients must be evaluated. Vitamin C has been observed to provide potential benefits against development of kidney disease. For example, vitamin C can help in controlling anemia in patients with chronic kidney disease, and it can have reno-protective effects since it improves the levels of urea, MDA, and catalase [110].

The brain consumes a large amount of glucose and oxygen, which indicates rapid metabolism with increased oxidative stress. Therefore, ascorbate is abundant in the brain tissue. Ascorbate accumulates in choroid plexus cells (CP) and then passes through the CSF into the brain [111]. More specifically, ascorbate enters CNS through SVCT2 present in CP on the basal side [112] of the CP and also probably through GLUT1 [113]. The brain steady-state ascorbate concentrations are remarkably high, and in addition, it also has exceptional retention of vitamin C in its tissues during states of deficiency. To this time, it unknown what the brain concentrations are as plasma ascorbate rises to pharmacological levels.

Regarding the nervous system, SARS-CoV-2 can have the capacity to cross the blood-brain barrier and cause multiple symptomatology in infected patients. Recent studies discovered that brain autopsies from COVID-19 patients had cortical neurons with viral load of SARS-CoV-2 [114]. Moreover, possible infection and minimal immune cell infiltrates were noted, providing evidence for SARS-CoV-2 neuroinvasive capacities. Regarding how vitamin C could play a role in the different manifestations of the nervous system, it has been seen that ascorbate can help in neuronal repair, myelination, and function [115]. Additionally, the review stated that vitamin C is an important antioxidant for the CNS since it scavenges ROS molecules, causing suppression of oxidative stress in the nervous system.

On the other hand, it has been deduced that one of the possibilities of neurological manifestations on COVID-19 patients is due to the excessive immune response and the cytokine storm [107]. Therefore, as established previously, it seems that the suppression or inhibition of the cytokine storm could help in preventing neuronal complications in addition to multiple organ manifestations in COVID-19 patients. As mentioned before, this could be done by providing HDIVVC since various studies have proven the different mechanisms of action this vitamin cofactor exerts regarding the cytokine storm.

## 11. Conclusions

Despite its favorable physiologic effects of ascorbate, which are both potent and multiple, it should not be considered as a sole treatment for SARS-CoV-2, but it does provide the immune system the necessary boost to combat the virus more efficiently, shorten the length of the disease, and prevent fatal complications. This important vitamin cofactor plays many roles in suppressing the pathological processes exerted by SARS-CoV-2. Vitamin C has immuno-supportive, anti-viral, anti-inflammatory, antioxidant, anti-thrombotic, and many other beneficial properties. Because it has all these capacities, it can be concluded that vitamin C, especially HDIVVC, can be seen as an important component in the treatment for SARS-CoV-2. What is also important to highlight is that in practically all of the pathophysiological stages of SARS-CoV-2, vitamin C can have a potential role in diminishing its detrimental effects. This is due to the pleiotropic physiological effects that ascorbate exhibits, such as inhibiting the cytokine storm, reducing oxidative stress, decreasing inflammation, and preventing thrombotic complications, among others. Moreover, vitamin C has the capacity to reduce mortality in COVID-19 patients while providing better clinical outcomes that do not include harsh side effects to the body. Furthermore, by supplementing vitamin C to COVID-19 patients, research states that it can reduce the high doses of medications, like corticosteroids. Vitamin C is a safe and low-cost option that should be contemplated as part of the treatment of COVID-19 patients.

On the other hand, vitamin C has been used as a treatment for many diseases, such as cancer, colds, inflammatory disorders, and others. Additionally, ascorbic acid is an important water-soluble molecule that is excreted very easily through urine. However, it can exert multiple beneficial physiological effects. COVID-19 patients usually possess low levels of vitamin C due to the stress imposed by SARS-CoV-2 infection. As a result, higher quantities of this vitamin cofactor are needed in order for the immune system to function properly during a viral attack. There is no doubt that the use of HDIVVC will be beneficial to virally infected patients. More research needs to be done to provide better clinical outcomes to COVID-19 patients. It is necessary to search for other available treatments that are less invasive yet capable of optimizing immune responses in infected COVID-19 patients. Vitamin C seems to be this additional component needed for a better treatment of COVID-19 patients.

## Data Availability

Not applicable.
